# Extensive Involvement of the Bilateral Trigeminal Nerve Branch as the Only Imaging Manifestation of IgG4‑Related Ophthalmic Disease

**DOI:** 10.5334/jbsr.3838

**Published:** 2025-03-03

**Authors:** Chendong He, Wei Yang

**Affiliations:** 1 Department of Radiology, Nanjing Hospital of Chinese Medicine, Nanjing 210022, Jiangsu, China; 2 Department of Radiology, Jiangsu Province Hospital of Chinese Medicine and Affiliated Hospital of Nanjing University of Chinese Medicine, Nanjing 210029, Jiangsu, China

**Keywords:** IgG4‑related ophthalmic disease, Trigeminal nerve, Magnetic resonance imaging

## Abstract

*Teaching point:* Immunoglobulin G4‑related ophthalmic disease (IgG4‑ROD) may present only as diffuse thickening of the orbital branches of the trigeminal nerve.

## Case Presentation

**Figure 1:**
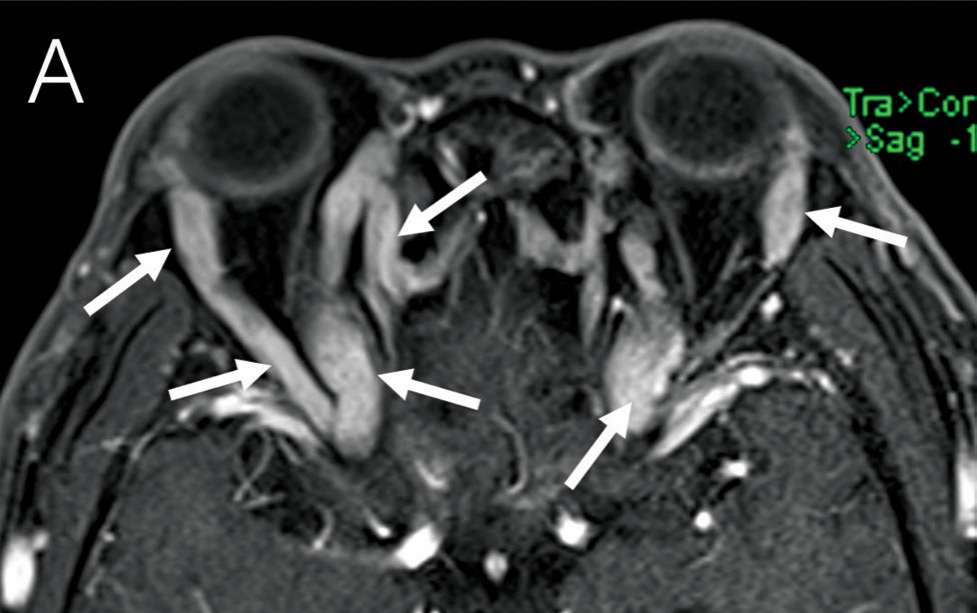

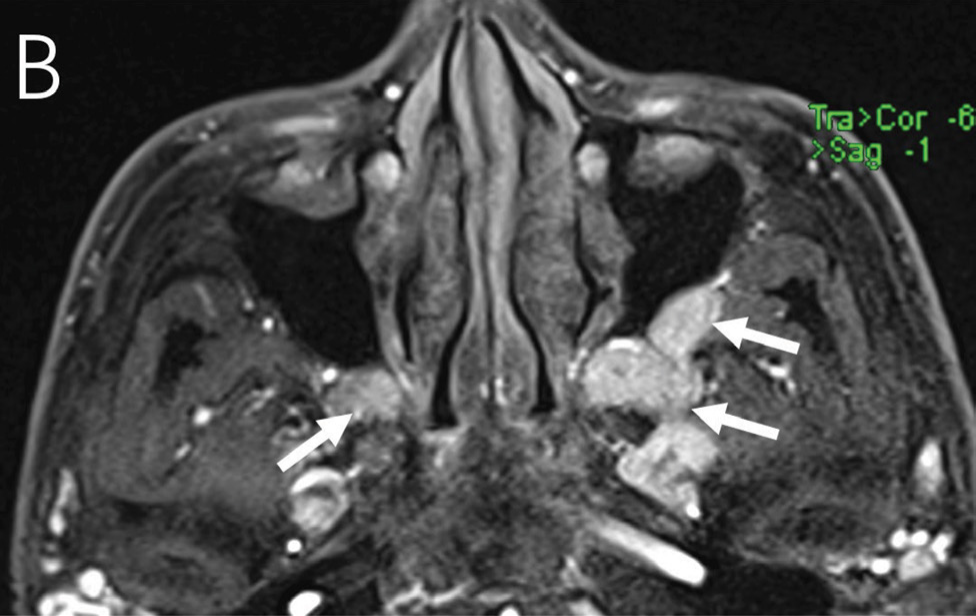

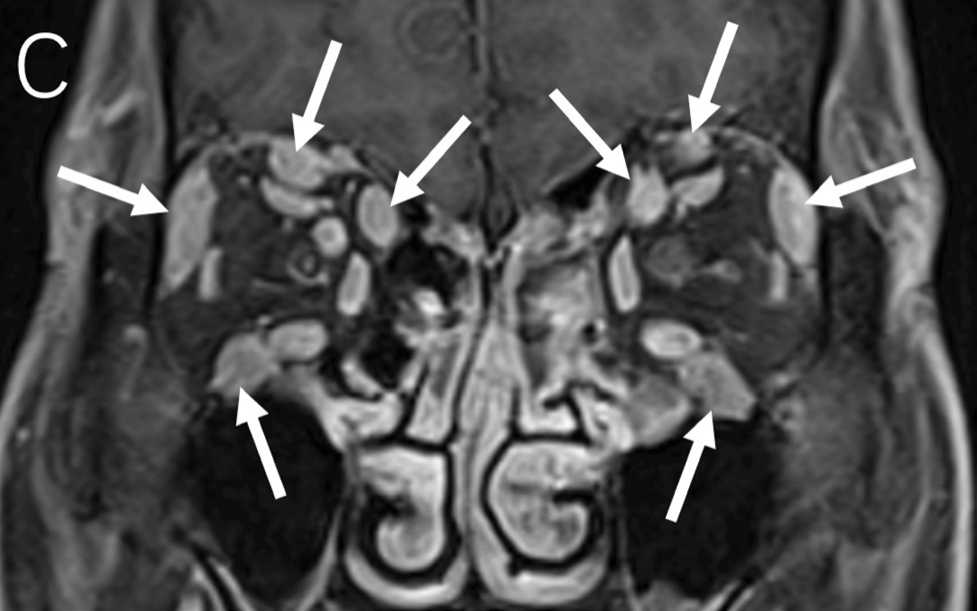

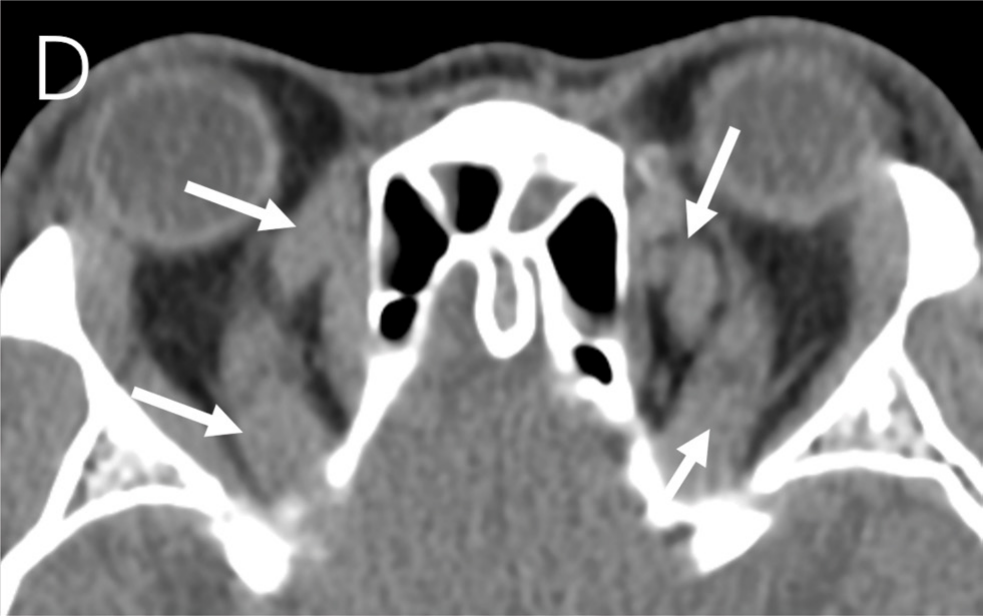

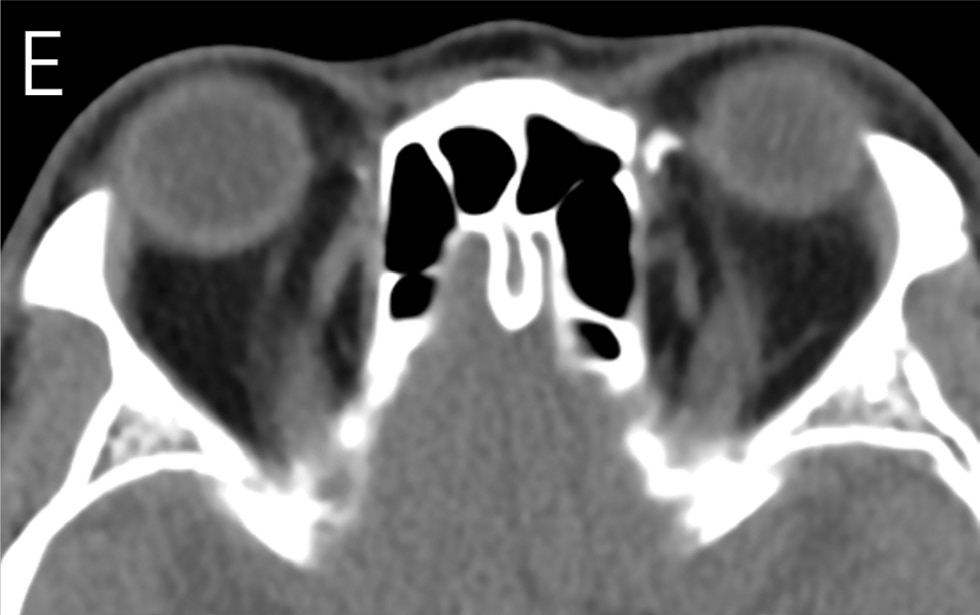

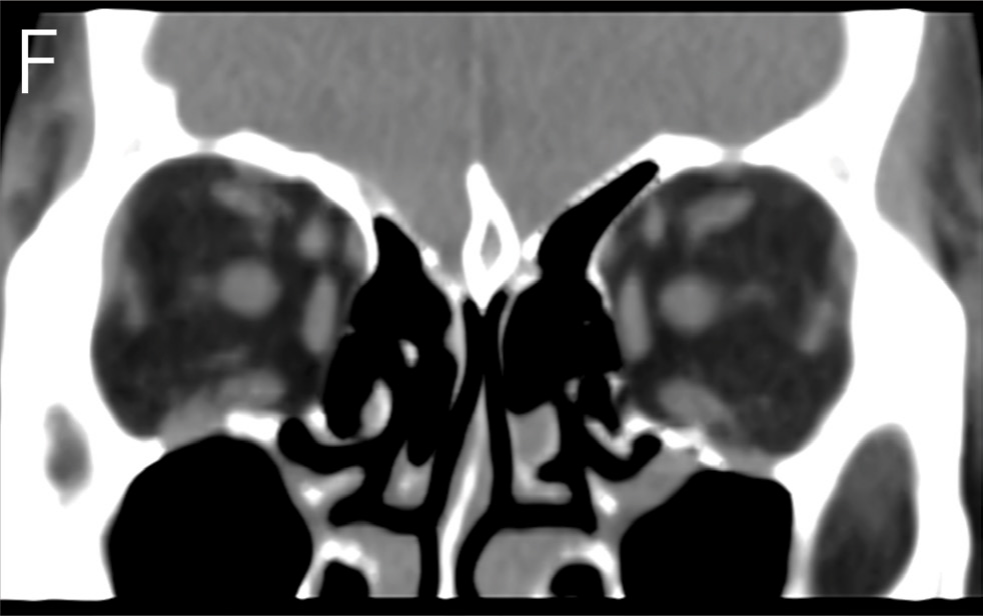
A 58‑year‑old male patient presented with bilateral eye protrusion for six months, blurred vision in the right eye for 15 days, and restricted eye movement. Axial **(A, B)**, Coronal **(C)** post‑contrast T1 fat‑saturated MRI and axial **(D)** CT showed tortuous and thickened periorbital nerve tissue (arrows). A axial **(E)** and coronal **(F)** orbital CT examination 4 months after hormone therapy showed that the lesion improved significantly.

A 58‑year‑old male patient complained of bilateral eye protrusion for 6 months, blurred vision in the right eye for 15 days, and restricted eye movement without pain in both eyes. Physical examination showed the absence of the light reflex in the right eye. Serological tests revealed an immunoglobulin G4 (IgG4) level of 38.07 g/L and an elevated absolute eosinophil count of 0.97×10^9^/L. Axial (Figure 1A, B) and coronal (Figure 1C) post‑contrast T1 fat‑saturated magnetic resonance imaging (MRI) and axial (Figure 1D) computed tomography (CT) showed extensive thickening with enhancement of the trigeminal nerve branches, localized compression of the lateral margin of the optic nerve, no significant thickening of the extraocular muscles, no enlargement of the lacrimal glands, and normal bilateral optic chiasm. The patient refused a biopsy; however, on the basis of the imaging findings and elevated serum IgG4 levels, we strongly suspected IgG4‑related ophthalmic disease (IgG4‑ROD). We administered anti‑inflammatory methylprednisolone intravenously, and upon reexamination, IgG4 levels decreased to 23.07 g/L. After treatment, the symptoms of protrusion symptoms improved, and the patient was discharged 1 week later. After discharge, the patient continued to take oral prednisone acetate, and 3 weeks later, a follow‑up test showed IgG4 had decreased to 12.40 g/L. Four months later, another follow‑up showed IgG4 at 9.91 g/L, and orbital CT showed significant improvement in the thickening of the orbital nerve tissue compared with before treatment (Figure 1E, F).

## Comments

Common symptoms of IgG4‑ROD include swelling and inflammation of the lacrimal glands, eyelids, and orbital tissues [1]. When IgG4‑ROD involves the orbital branches of the trigeminal nerve, inflammation can extend along the nerves and spread to the surrounding area [1, 2]. This can result in pain, numbness, or even ophthalmoplegia. In severe cases, vision can be affected owingto the involvement of the optic nerve or other critical structures. Diagnosing IgG4‑ROD involves a combination of radiological, histopathological, and serological criteria. Imaging shows characteristic findings, such as enlargement of the lacrimal gland, extraocular muscles, or orbital soft tissue masses. Histopathological examination shows dense infiltration by lymphocytes and plasma cells, sometimes with storiform fibrosis and obliterative phlebitis. Immunohistochemical staining shows more than 10 IgG4+ plasma cells per high‑power field and an IgG4+/IgG+ cell ratio greater than 40%. Serum IgG4 concentrations are often elevated (above 135 mg/dL). The diagnosis can be challenging because its manifestations can mimic other inflammatory, infectious, and neoplastic conditions. Several clinical studies have demonstrated that trigeminal nerve enlargement can occur not only in IgG4‑ROD but also in other orbital lymphoproliferative disorders. Hiroshi Goto and colleagues reported that, among 149 IgG4‑ROD cases, 35 (23.5%) exhibited trigeminal nerve enlargement, compared with 7 (3.2%) of 218 lymphoma cases. Although the incidence of trigeminal nerve enlargement is higher in IgG4‑ROD, the possibility of lymphoma should not be disregarded [1]. Corticosteroids are the preferred treatment to reduce inflammation. For patients who do not respond to steroids or need long‑term management, drugs such as rituximab or azathioprine may be used. In some cases, surgical intervention may be required to relieve symptoms or obtain a diagnostic biopsy.

In conclusion, involvement of the orbital trigeminal nerve branches in IgG4‑ROD can lead to significant symptoms affecting sensory and, less commonly, motor functions around the eye. Prompt diagnosis and treatment are crucial to manage these symptoms effectively. With appropriate treatment, many patients experience significant improvement. However, the disease can recur and may require long‑term management to prevent relapses.

## References

[r1] Goto H, Sone K, Asakage M, Umazume K, Usui Y, Mori H. Evaluation of the specificity of trigeminal nerve enlargement in the diagnosis of IgG4‑related ophthalmic disease. Jpn J Ophthalmol. 2024 November;68(6):676–680. 10.1007/s10384-024-01116-9. 39312048

[r2] Elkhamary SM, Cruz AAV, Zotin MC, et al. Involvement of multiple trigeminal nerve branches in IgG4‑related orbital disease. Ophthalmic Plast Reconstr Surg. 2021 March‑April 01; 37(2):176–178. 10.1097/IOP.0000000000001733. 32501880

